# A horizon scan of music therapy in education

**DOI:** 10.3389/fpsyg.2025.1635258

**Published:** 2025-12-05

**Authors:** Yifei Lyu, Xiangyu Wang

**Affiliations:** 1 School of Music, Baotou Teachers’ College, Inner Mongolia University of Science and Technology, Baotou, China; 2 School of Architecture and Art Design, Inner Mongolia University of Science and Technology, Baotou, China

**Keywords:** music therapy, music education, emotional well-being, content analysis, bibliometric analysis

## Abstract

**Introduction:**

Music therapy has gained increasing recognition for its benefits in educational settings, particularly in supporting students with diverse learning needs and promoting overall well-being. However, existing reviews reveal several limitations, including fragmented research efforts, limited interdisciplinary and international collaboration, and practical constraints such as insufficient funding and a shortage of trained music therapists. These gaps highlight the need for a more comprehensive understanding of the field’s development.

**Methods:**

This research systematically examined publication trends, key contributors, and thematic clusters in the field of music therapy in education. Both quantitative indicators (e.g., publication and citation patterns) and qualitative thematic analysis were employed to generate an integrated overview of current research dynamics.

**Results:**

The analysis revealed a strong increase in publications and citations, largely driven by Western institutions. Despite this growth, several persistent challenges were identified: research fragmentation, limited international collaboration, and a notable underrepresentation of studies focusing on media-related aspects of music therapy.

**Discussion:**

The study recommends strengthening interdisciplinary and international research networks, expanding investigations into media-related practices, developing standardized assessment tools, and increasing support in terms of funding and professional development. These recommendations provide a strategic roadmap for advancing music therapy in educational contexts and offer actionable guidance for researchers, educators, and policymakers aiming to enhance its integration and effectiveness.

## 1 Introduction

Music therapy has become vital in educational settings, particularly for children with special needs. Music therapy improves learning outcomes and emotional well-being and has a long history and is backed by a number of theoretical models. From its early promotion by organizations like the National Society of Musical Therapeutics in the early 20th century to its integration into modern educational frameworks, music therapy has proven effective in addressing language, behavioral, and social challenges among students with neurological conditions such as autism spectrum disorder (Riley et al., 2019). Music therapy is a vital tool for both educators and therapists because it not only supports social interaction and inclusivity but also cognitive and emotional development.

Despite its significant benefits, existing literature reviews on music therapy in education reveal critical gaps and limitations. Current studies often present fragmented research efforts, with isolated thematic clusters and limited interdisciplinary collaboration, which hinder the development of cohesive theoretical frameworks. Furthermore, bibliometric analyses are crucial for charting research trends, evaluating contributions from around the world, and identifying influential studies, yet they have been conspicuously lacking in this field. The lack of comprehensive bibliometric reviews limits our understanding of the research landscape, collaboration networks, and the overall impact of music therapy in education. Addressing these gaps through a bibliometric approach is crucial, as it provides a structured and quantitative assessment of the existing literature, facilitating informed decision-making and strategic planning for future research.

This study aims to conduct a bibliometric and content analysis of international research on music therapy in education in the past decade. It uses the bibliometrix package in R to map research trends, assess the productivity and impact of important sources and affiliations, and investigate international collaboration networks using data from the Web of Science and Scopus databases. This study also provides a detailed roadmap of the research output, identifies emerging trends, and highlights influential studies that can guide future investigations in the field.

The main contributions of this study are as follows:

(1) Identifying and visualizing the evolution of key themes and methodologies in music therapy within educational settings over the past two decades.
(2) Evaluating the extent and impact of international collaborations, highlighting key contributing countries and institutions.
(3) Determining the most impactful journals and publications offers insights into disseminating and recognizing research findings.
(4) Providing actionable recommendations to address identified challenges, promote interdisciplinary and international collaborations, and enhance the overall effectiveness of music therapy practices in education.

## 2 Methodology

**Figure 1:**
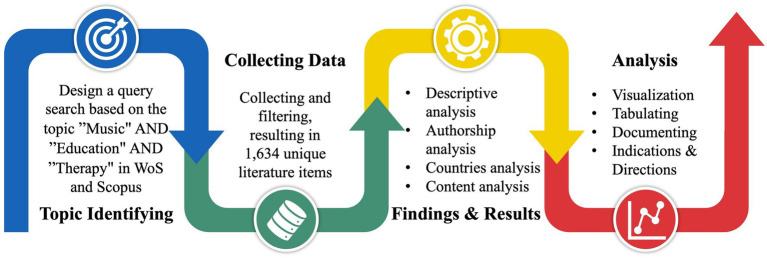
Methodology of the study.

Assessing and analyzing the current state of Music Therapy in Education research motivates this bibliometric and content analysis study. These analyses are able to identify research questions, limitations, methodologies, and knowledge boundaries, providing a comprehensive roadmap for future investigations (Altarturi et al., 2020). The bibliometric approach employed in this study involves four key phases: designing search keywords, collecting and creating the dataset, outlining findings, and analyzing the results, as shown in Figure 1. These phases help define relevant research areas, uncover current trends, and highlight influential studies that guide further research in the field.

The literature collection was conducted in January 2025 using the Web of Science (WoS) and Scopus databases. These were chosen because they cover a lot of ground and can produce bibliometric data. WoS is known for having a lot of high-impact publications, while Scopus has the biggest database of peer-reviewed research in many fields (Altarturi et al., 2023a). Using a structured query with Boolean operators, the study incorporated keywords related to music therapy and education, including synonyms, to ensure a broad and inclusive search. The search query combined keyword groups related to music therapy and education. By using Boolean operators (“AND,” “OR”), it captures all relevant variants such as “music therapy,” “music-based intervention,” “music education,” “school,” and “learning.” The query collected publications from 2004 to 2024 and retrieved 1,463 records from Scopus and 794 from Web of Science (WoS). Using the mergeDbSources() function of the bibliometrix R library (as described in its official documentation) to automatically remove duplicates, followed by a manual screening of titles and abstracts for accuracy, the final dataset comprised 1,634 unique documents.

For the bibliometric analysis, the study utilised the bibliometrix package in R accessed through the Biblioshiny web interface. Biblioshiny facilitates comprehensive data processing. This software supports various analytical tasks such as content analysis, corpus pre-processing, and visualization of findings. We used the package’s standard functions [e.g., *biblioAnalysis(), conceptualStructure(), thematicMap()*] to do all the analysis, including keyword co-occurrence, thematic evolution, and conceptual structure mapping. We followed the package’s official documentation where theme extraction and clustering were done automatically, making sure that the results were consistent and could be repeated without the need for manual coding or inter-coder validation. Furthermore, country-level analysis was predicated on the institutional affiliation of the corresponding author, as cataloged in Scopus and Web of Science, instead of the researcher’s individual nationality.

Alongside the bibliometric analysis, a narrative synthesis was performed to qualitatively assess theoretical frameworks, therapeutic mechanisms, and educational applications of music therapy. The narrative synthesis was directly extracted from the identical bibliometric corpus, guaranteeing methodological coherence between quantitative and qualitative analyses. From the 1,634 documents, a subset of 212 publications was manually screened based on inclusion criteria emphasizing (a) educational or school-based contexts, (b) music therapy models such as CoMT, NMT, or Dalcroze, and (c) empirical or theoretical contributions relevant to learning or psychological outcomes. Papers without explicit educational components or insufficient methodological detail were excluded. While no formal quality appraisal was conducted, consistent with bibliometric standards, only peer-reviewed and conceptually relevant works were included to ensure rigor. The two analyses complemented each other: the bibliometric mapping quantified global patterns and trends, while the narrative synthesis contextualized these results by interpreting theoretical foundations, intervention mechanisms, and pedagogical applications within education.

## 3 Findings

### 3.1 Descriptive analysis

The Summary Information section provides essential statistics on the examined dataset. Table 1 highlights 1,634 items published between 2004 and 2024 across 937 sources, involving 5,064 authors. A co-author ratio of 3.61 and an international collaboration rate of 6.18% were recorded. Keywords Plus (6,565) and author’s keywords (3,763) show diverse research focus, with 14.17 average citations per document. A 9.43% annual growth rate indicates rising interest and development in music therapy in education. Additionally,

**Table 1:** Summary information of the dataset.

Description of the dataset	Value
Timespan	2004:2024
Sources (Journals, Books, Conferences, others)	937
Documents	1,634
Annual Growth Rate %	9.43
Average citations per document	14.17
Keywords Plus	6,565
Author’s Keywords	3,763
Authors	5,064
Authors of single-authored docs	366
Single-authored documents	415
Co-Authors per Documents	3.61
International co-authorships %	6.181

Table 2 reveals a predominant reliance on journal articles, constituting over two-thirds of the dataset.

**Table 2:** Types of literature items in the dataset.

Description of the dataset	Value	% of dataset
Article	1,114	68.2%
Review	266	16.3%
Proceedings paper	57	3.5%
Book chapter	49	3.0%
Book	34	2.1%
Conference paper	28	1.7%
Editorial	24	1.5%
Meeting abstract	21	1.3%
Others (note, letter, short survey, book review, and erratum)	41	2.5%

#### 3.1.1 Annual growth of publications

**Figure 2:**
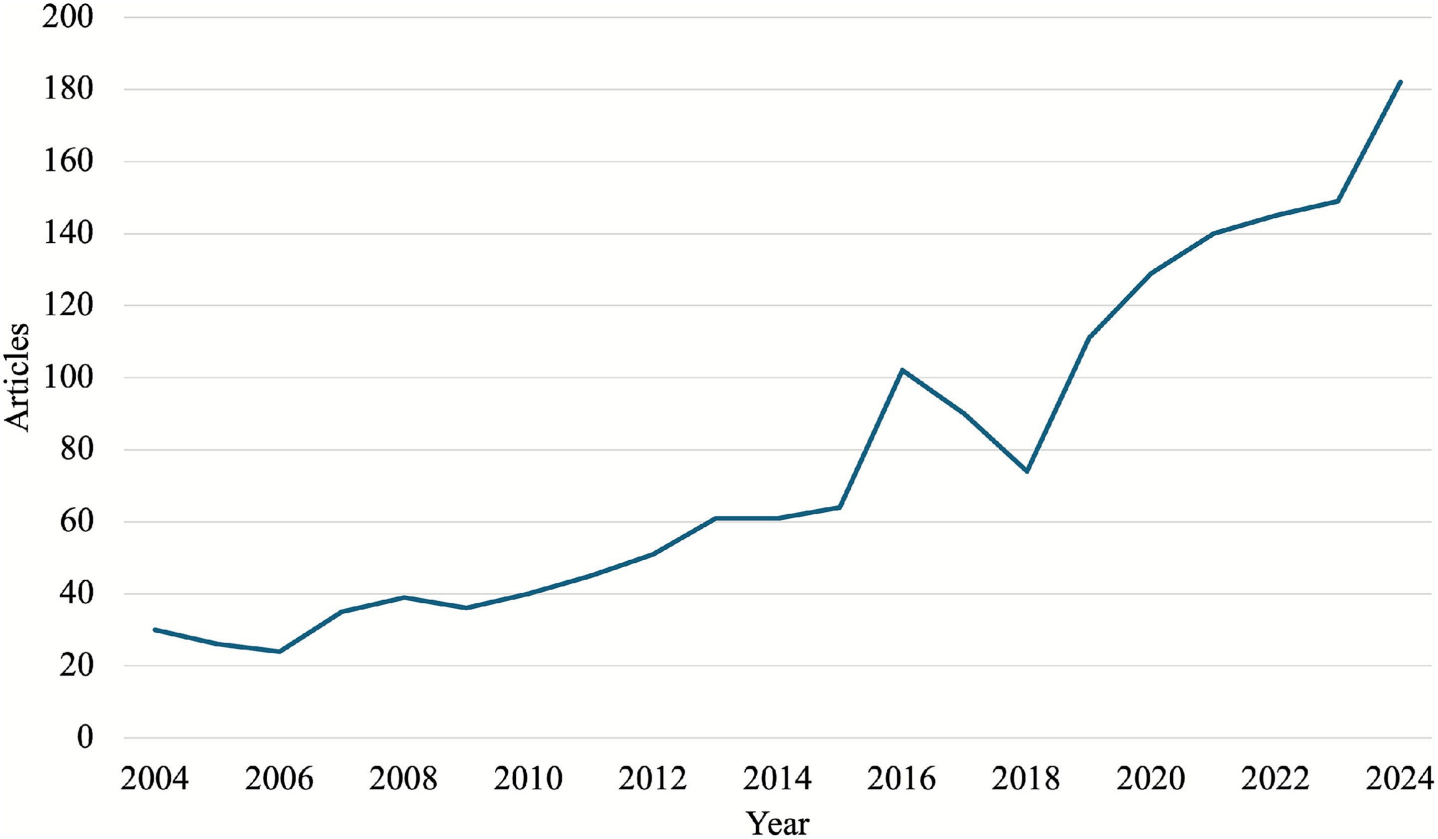
Yearly publication growth for the ethnic media domain.

The annual growth in Music Therapy in Education research publications (shown in Figure 2) demonstrates a consistent upward trajectory, increasing from 30 articles in 2004 to 182 in 2024. The significant acceleration in publication volume post-2015 likely reflects enhanced institutional support and a growing acknowledgment of music therapy’s impact on student outcomes.

#### 3.1.2 Top 15 impacting sources

The impact and productivity of scientific sources in Music Therapy in Education research are assessed through key bibliometric indicators such as the number of publications (NP), total citations (TC), and h-index.

Table 3 highlights the most influential journals contributing to the field, providing a comprehensive overview of the primary platforms shaping current discourse. These metrics collectively offer insights into the journals’ ability to disseminate impactful research and sustain academic relevance over time.

**Table 3:** Analysis of the top 15 most impacting sources in the dataset.

Source	NP	TC	h-index	PYS
Music Therapy Perspectives	70	271	9	2011
Journal of Music Therapy	56	692	15	2004
Arts In Psychotherapy	46	458	13	2004
Nordic Journal of Music Therapy	38	183	8	2007
International Journal of Environmental Research and Public Health	17	201	6	2016
Psychology Of Music	17	119	7	2004
International Journal of Community Music	16	79	5	2010
Plos One	11	363	7	2013
Frontiers In Psychology	11	362	9	2014
International Journal of Music Education	11	65	5	2005
Journal of Autism and Developmental Disorders	10	879	8	2006
Cochrane Database of Systematic Reviews	10	424	8	2008
Supportive Care in Cancer	9	163	6	2015
Complementary Therapies in Medicine	8	185	7	2005
Journal of Alternative and Complementary Medicine	8	145	5	2007

Among the top sources, the Journal of Autism and Developmental Disorders stands out with the highest total citations. Music Therapy Perspectives and the Journal of Music Therapy also demonstrate substantial influence. Additionally, interdisciplinary journals like Plos One and Frontiers in Psychology indicate the field’s expanding reach and integration with broader psychological and community music research.

Despite the presence of highly cited journals, the data reveals a concentration of impactful work within a limited number of sources. A large portion of the 937 sources contributing only a single publication and a notable number receiving no citations. The emergence of 105 new sources in 2023 onwards suggests growing diversification.

#### 3.1.3 Top contributing affiliations

**Figure 3:**
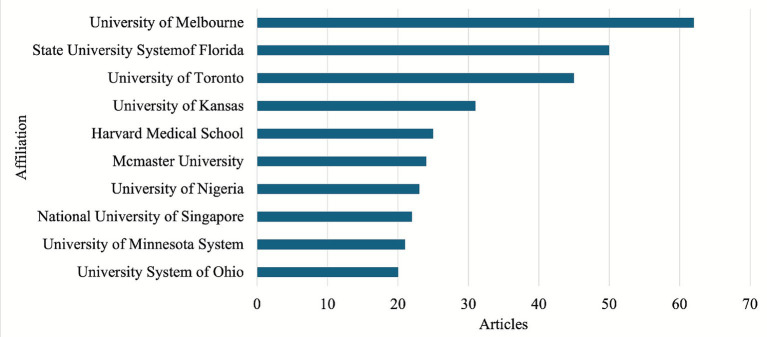
Top contributing affiliations based on the number of articles.

The number of publications from affiliation is a crucial metric to gauge its academic influence and contribution to Music Therapy in Education research. Figure 3 highlights the leading institutions, with the top 10 affiliations collectively accounting for a significant portion of the research output. Institutional names were standardized to merge duplicates (e.g., ‘University of Melbourne’ and ‘The University of Melbourne’) and aggregated by system when applicable (e.g., University of Minnesota System) Notably, universities from diverse geographical regions, including North America, Asia, and Africa, demonstrate substantial involvement, reflecting the global interest and varied perspectives contributing to the field’s advancement.

**Figure 4:**
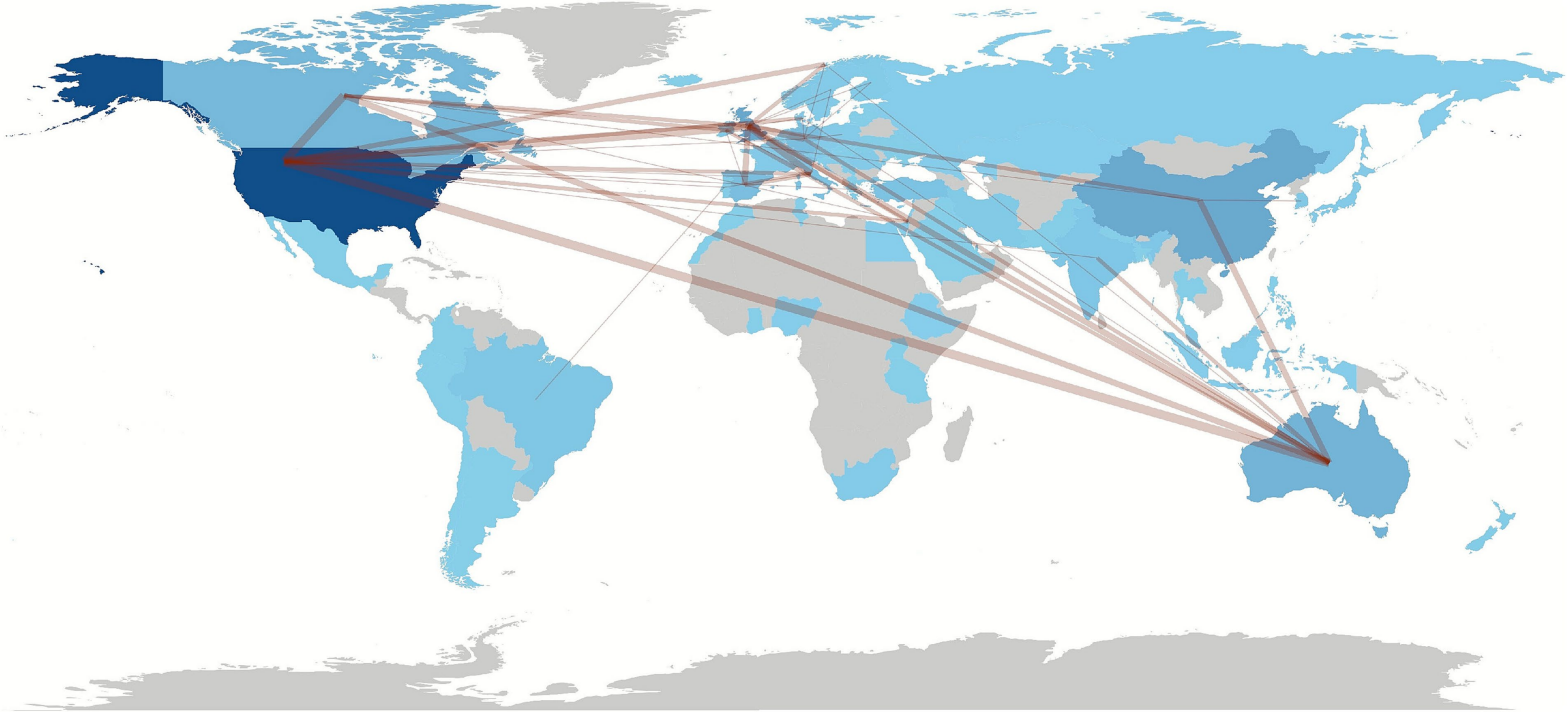
Countries’ collaboration on the ethnic media field.

Despite the prominent contributions from these key institutions, the data reveals that most affiliations have limited engagement, with nearly half contributing only to a single publication. Enhancing collaboration and supporting a wider range of institutions could foster a more inclusive and comprehensive development of music therapy practices in educational settings (Figure 4).

### 3.2 Countries analysis

The landscape of ethnic media research is shaped by contributions from various countries. In the dataset under analysis, 75 countries have made their mark. Evaluating the contributions of each country offers a panoramic view of the global research dynamics, highlighting the influence and commitment of different nations to this domain. This section delves into two distinct metrics: countries’ productivity and collaborations.

#### 3.2.1 Countries’ impact and productivity

In this section, each document is attributed to the country of its primary author, ensuring that every document is associated with just one country. This method facilitates the calculation of Single Country Publications (SCP) and Multiple Country Publications (MCP) metrics, along with the Total Citation (TC), number of publications, and frequency appearance in all literature. The MCP ratio further refines our understanding of collaboration levels among countries. Additionally, this section delves into the total citation counts for each nation, as detailed in Table 5.

The United States remains the foremost contributor, demonstrating both high productivity and substantial citation impact. Other significant contributors include China, the United Kingdom, Australia, and Canada. Notably, countries like Italy and the United Kingdom show higher MCP percentages.

Despite the strong contributions from these leading nations, the data reveals that the majority of countries engage primarily in single-country publications, with limited international collaboration. Furthermore, while most countries have made multiple contributions, a significant number still have minimal participation, highlighting disparities in research resources and collaborative networks.

#### 3.2.2 Collaboration between countries

This section examines the international collaboration dynamics within Music Therapy in Education research, highlighting the patterns of co-authorship between countries, as shown in Table 4. The collaboration network is predominantly centered around a few key partnerships, with the USA and the United Kingdom leading the way through extensive joint publications. Strong ties also exist between the USA and Australia, as well as the USA and Canada, reflecting a significant concentration of collaborative efforts among these nations. Additionally, European countries such as Germany, Italy, and Spain demonstrate notable collaborative relationships, often interconnected with the United Kingdom and Australia, suggesting regional clusters of cooperative research.

**Table 4:** Analysis of countries’ impact and productivity.

Country	No. of publication	Freq. appearance	SCP	MCP	TC
USA	421	883	402	19	6,533
China	131	214	121	10	835
United Kingdom	97	198	85	12	2,291
Australia	92	175	80	12	1943
Canada	71	122	66	5	1843
Spain	50	141	48	2	217
Germany	46	102	43	3	551
Turkey	40	62	40	0	334
Italy	35	82	29	6	660
Brazil	26	43	25	1	330

Despite these active partnerships, the overall level of international collaboration remains relatively limited, with most country pairs engaging in only a handful of joint publications. Furthermore, the emergence of collaborations involving countries like China, India, and Brazil indicates gradual expansion, yet these remain sparse compared to established partnerships.

### 3.3 Authorship analysis

Various metrics, including the h-index, total citation count (TC), and number of publications, are utilised to evaluate authors’ scientific output and influence in Music Therapy in Education research, as shown in Table 5.

**Table 5:** Authorship analysis based on publication metrics.

Author	NP	h_index	Fraction	TC	PYS
Michael J Silverman	21	8	16.20	183	2007
Felicity Baker	12	7	5.81	180	2007
Katrina Skewes McFerran	12	8	5.58	187	2004
Lori F Gooding	10	4	5.00	59	2013
Eugenia Hernandez-Ruiz	8	3	4.33	46	2017
Abbey L Dvorak	7	4	2.48	52	2017
Kate E Williams	7	6	2.27	222	2008
Anthony Meadows	6	2	2.07	7	2020
Adam Ockelford	6	3	4.25	34	2008
Daphne Rickson	6	5	3.45	73	2012

Prominent contributors such as Michael J. Silverman and Felicity Baker exhibit substantial publication records and citation impacts. However, the analysis reveals that most authors engage only once, highlighting a highly fragmented authorship landscape.

The predominance of single-publication authors and the many works with minimal citations indicate challenges in achieving widespread recognition and impact. Additionally, the recent influx of new contributors points to growing interest, yet the field continues to rely heavily on a small group of prolific authors. Enhancing support for emerging scholars and promoting sustained contributions can help ensure that Music Therapy in Education benefits from both breadth and depth of scholarly input.

### 3.4 Content analysis

#### 3.4.1 Author keywords

**Figure 5:**
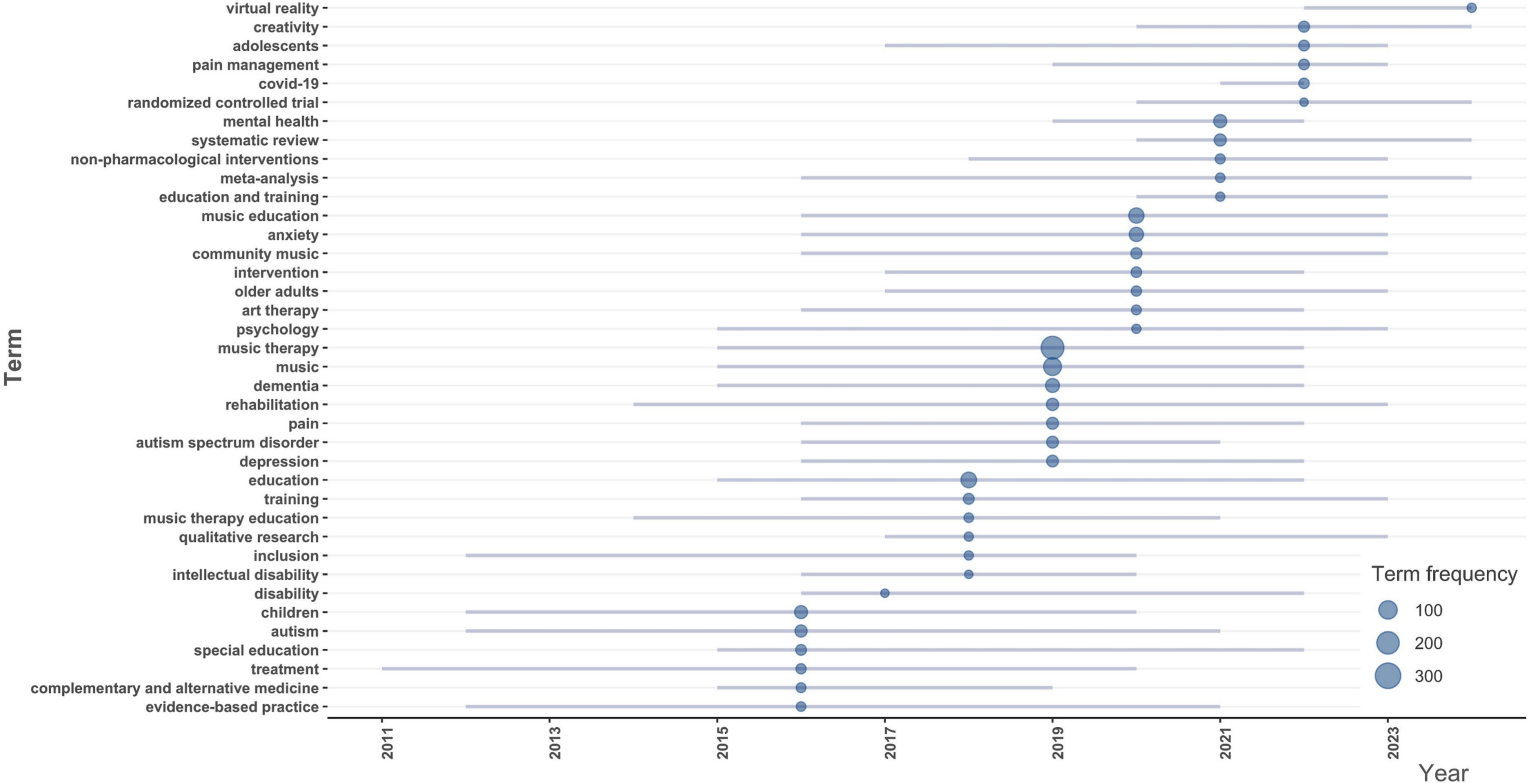
Trend Topics over time in the field of music therapy education.

This section uses author keywords to reveal significant trends in Music Therapy in Education research. The Timeline Bubble Plot, illustrated in Figure 5, highlights shifts in focus, with early studies focusing on foundational themes like music therapy and education. Over time, specialised topics such as mental health, anxiety, and dementia emerged, reflecting the field’s growth in addressing psychological and developmental challenges.

From 2015 onwards, core themes like “music therapy” and “music” remained prominent, while emerging topics like “virtual reality,” “creativity,” and “randomised controlled trial” signal the integration of innovative methodologies. The rise of “covid-19” highlights responsiveness to global health crises, alongside increasing focus on holistic and evidence-based mental health and education approaches.

#### 3.4.2 Keywords plus

**Figure 6:**
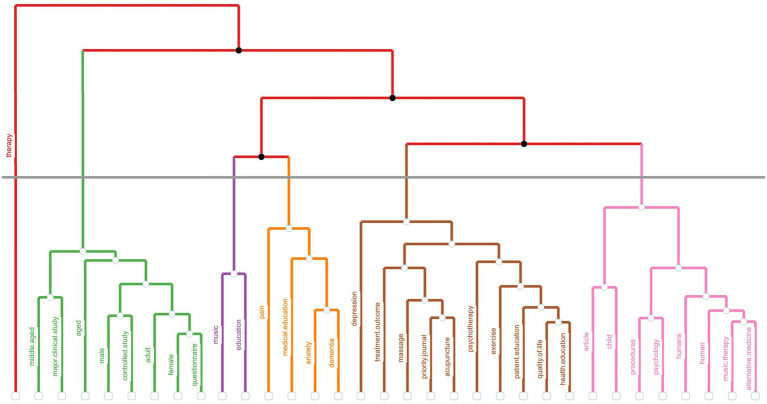
Dendrogram of the Keywords Plus in the field of music therapy education.

This section uses Keywords Plus with Multiple Correspondence Analysis (MCA) to identify thematic clusters in Music Therapy in Education research, illustrated in Figure 6. Six clusters emerge, with Cluster 1 focusing on foundational concepts like “music therapy” and “psychology,” Cluster 2 addressing demographic and methodological aspects such as “female,” “male,” and “controlled study,” and Cluster 3 highlighting the intersection of “music” and “education.”

Clusters 5 and 6 focus on health-related themes, including conditions like “anxiety,” “dementia,” and “pain,” as well as broader topics like “quality of life” and therapeutic interventions such as “psychotherapy.” Cluster 4, represented by “therapy,” is a central link between various therapeutic approaches.

#### 3.4.3 Abstract and title analysis

**Figure 7:**
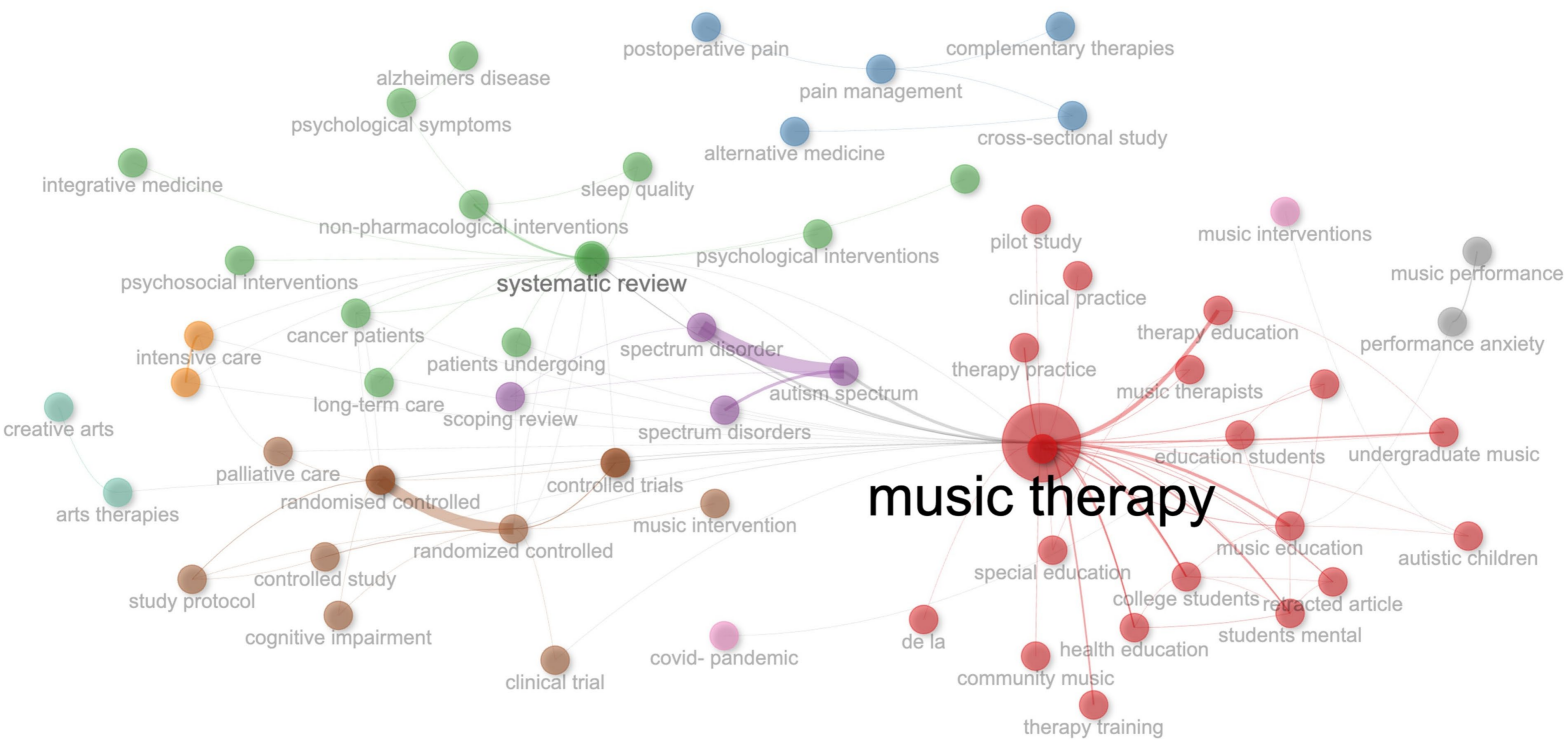
Network co-occurrence illustration for title and abstract contents in the field of music therapy education.

This section analyses abstracts and titles using Natural Language Processing (NLP) techniques (Altarturi et al., 2023b), focusing on bigrams and co-occurrence patterns to uncover key research themes in Music Therapy in Education. The Walktrap clustering algorithm identifies six thematic clusters, highlighting interconnected concepts and revealing research strengths and gaps, as shown in Figure 7.

Cluster 1 focuses on foundational elements like “music therapy” and “psychology,” while Cluster 2 addresses demographics and methodologies such as “controlled study.” Cluster 3 emphasises the role of “music” in “education,” and Clusters 5 and 6 explore health themes like “anxiety” and “pain management,” showcasing music therapy’s application in diverse health contexts.

## 4 Music therapy in education

Music therapy has become a vital component in educational settings, particularly for children with special needs. Its application is grounded in a rich historical context and supported by various theoretical models that underscore its effectiveness in enhancing learning outcomes and emotional well-being. This section explores the theoretical frameworks, benefits, methodologies, and challenges of music therapy in education.

To avoid conceptual overlap, this study distinguishes music therapy from music education. Music therapy is a clinical and evidence-based practice conducted by certified therapists to achieve therapeutic, emotional, or cognitive goals, as American Music Therapy Association clarified. In comparison, music education focuses on developing musical skills, creativity, and literacy through structured pedagogy. While both share musical processes and learning contexts, their objectives, methods, and outcomes differ, with therapy targeting personal well-being and education emphasizing artistic and academic development.

### 4.1 Theoretical framework

Music therapy has a long history, beginning with early mentions in 1789 and evolving through formal academic programs in the mid-20th century under pioneers like E. Thayer Gaston. It has become particularly beneficial in educational settings, enhancing language, behavioral, and social skills for students with neurological challenges and autism (Riley et al., 2019).

#### 4.1.1 Developmental models

One of the important models focuses on students’ growth and development in skills through music. Music education fosters cognitive development by enhancing perception, learning, and memory skills. It also supports emotional growth by allowing students to express and manage emotions through music (Hargreaves and Lamont, 2017). For example, the Sequential Development model outlines several steps, such as personal connections and growth. The student who engages with music while learning a particular skill will develop that skill more effectively (Dvorak et al., 2017).

#### 4.1.2 Ecological models

Environmental influences on the students are considered in the Ecological model, using theories like Bronfenbrenner’s Ecological systems theory. This is the immediate environment where students interact directly, such as family, school, and peers. Teachers and parents play a crucial role in this system by providing direct support and creating a nurturing environment (Khairul Amali et al., 2023). The relationship between home and school enables collaboration and communication between teachers and parents for the benefit of the children. Moreover, a comprehensive framework between home, school, community, and virtual support can optimise students’ learning experiences (Olson et al., 2023).

#### 4.1.3 Community music therapy

Another model is Community Music Therapy (CoMT), which emphasises social interaction and community involvement. These models are encouraged to be used in communities like schools, as CoMT can help students with social challenges by involving them in group music activities, fostering social skills, and building relationships. Some studies show that CoMT children with autism spectrum disorder (ASD) particularly benefit from social interaction in comparison with traditional music education (Wang and Zhang, 2023). Other studies indicated that CoMT is linked to critical theory and conflict transformation, which is beneficial in school settings to promote harmony among students (Hassanein, 2023).

#### 4.1.4 Neurological models

Neurological models demonstrate that music therapy stimulates brain areas linked to language, memory, and emotion, improving cognitive functions and brain connectivity in students with disabilities or ADHD. This approach allows for tailored interventions that enhance learning outcomes (Lai et al., 2023).

#### 4.1.5 Integrative models

Integrative models combine elements from various theories, such as the Biopsychosocial Model, addressing biological, psychological, and social factors. In education, these models enhance learning outcomes by connecting language skills, content, culture, and technology (Qizi, 2024).

### 4.2 Benefits of music therapy in education

In the following section, the study will discuss the emotional and psychological benefits, cognitive benefits, and socio-emotional benefits of musical therapy in education and the strategies for implementing it in educational settings.

#### 4.2.1 Emotional and psychological benefits

Music therapy offers a safe and creative outlet for emotional expression, enhancing emotional intelligence and reducing behavioral issues in the classroom (Wang et al., 2022). It effectively manages anxiety, depression, and trauma, thereby improving overall well-being and quality of life, as evidenced by significant reductions in psychological distress and improved mood regulation in structured programs (Chen et al., 2024).

#### 4.2.2 Cognitive benefits

Music therapy enhances attention, concentration, and executive functions, particularly benefiting students with ADHD by reducing hyperactivity and improving focus (Lee et al., 2024). Additionally, it improves memory retention and recall by engaging both hemispheres of the brain, supporting learning in subjects like mathematics and language arts, and fostering creativity and critical thinking skills (Kousloglou et al., 2023; Nabila et al., 2024).

#### 4.2.3 Socio-emotional benefits

Music therapy cultivates social skills and empathy through group activities such as composition and improvisation, promoting pro-social behaviors and emotional expression (Silke et al., 2024). It also boosts self-esteem and confidence, particularly in children with ADHD and other disabilities, fostering a growth mindset and motivation to face challenges (Sholeh and Supena, 2021).

#### 4.2.4 Implementation of strategies in educational settings

Integrating music therapy into school curricula requires close collaboration between educators and certified music therapists, along with tailored interventions and professional development for teachers (Marcos Treceño and Arias Gago, 2024). Technological integration enhances accessibility and engagement, while supportive school environments and dedicated spaces for music activities significantly improve students’ academic performance and emotional health (Ahmed and Mohammed, 2024).

### 4.3 Methodologies in music therapy

Various music therapy methodologies address the needs of students with special educational requirements, enhancing emotional, cognitive, social, and behavioral functioning. Integrating these approaches into the curriculum creates inclusive learning environments.

#### 4.3.1 Individualised music interventions

Personalised music interventions aligned with students’ Individualised Education Program (IEP) goals improve communication, social skills, and emotional expression in conditions like ASD and ADHD. Music therapy enhances emotional management and social development (Marcos Treceño and Arias Gago, 2024).

#### 4.3.2 Group music therapy sessions

Group music therapy significantly enhances social Group settings foster social interaction, emotional health, and empathy. These sessions enhance social skills for children with ASD and adolescents with depression (Choi and Kirby, 2023). Improve interpersonal relationships and self-expression, and facilitate group music-making that leads to notable social development gains.

#### 4.3.3 Music as a tool for behavioral management

Integrating music into daily routines supports emotional regulation, encourages positive behaviors, and refines classroom dynamics. Techniques like Musical Contour Regulation Facilitation (MCRF) aid emotion regulation in early childhood (Sena Moore and Hanson-Abromeit, 2015). Coordinated rhythmic movements improve focus, self-regulation, and overall classroom behavior (Williams, 2018).

#### 4.3.4 Integration with educational curriculum

Embedding music therapy within academic subjects like language arts, math, and science creates holistic learning experiences and enhances student engagement. This interdisciplinary approach promotes critical and creative thinking skills necessary for 21st-century learners (Kuwar and Acharya, 2024). In math, music integration improves cognitive and motor skills (Souto, 2024). Educational music therapy advances inclusion, emotional management, and social development, ultimately improving learning outcomes (Marcos Treceño and Arias Gago, 2024).

#### 4.3.5 Professional development for educators

Training in music therapy techniques empowers educators to create inclusive and supportive classrooms, ensuring effective application of music-based interventions (Li, 2024). Comprehensive professional development, including hands-on training and innovative tools like virtual reality, enhances teachers’ technical and pedagogical competence, addressing challenges such as limited resources (Elhambakhsh et al., 2024).

## 5 Challenges and limitations

The study highlights significant challenges in the field of Music Therapy in Education. Research is fragmented, with thematic clusters operating in isolation, limiting the development of cohesive theoretical frameworks. Interdisciplinary integration remains inadequate, as methodological approaches are not consistently linked to health or educational outcomes. Underrepresentation of media practices and cultural perspectives further constrains the field, while Western-centric viewpoints restrict global applicability. Oversaturation in core areas like anxiety and music therapy may stifle innovation.

Practical implementation in schools also faces substantial barriers. A shortage of qualified music therapists (Springer and Gooding, 2023) and misconceptions among educators and administrators hinder effective integration. Limited resources, budget constraints (Xinyue, 2024), and time restrictions (Clements-Cortes, 2023). impede consistent music therapy sessions. Individual variability in student responses, a lack of standardised assessments, and insufficient training opportunities (Raposo et al., 2023). make evaluation and advocacy more challenging. Addressing these issues through enhanced support, interdisciplinary collaboration, and broader cultural perspectives is essential for realizing the full potential of music therapy in education.

## 6 Discussion and future directions

The bibliometric and content analyses collectively highlight both the advancement and structural challenges within Music Therapy in Education. The field has experienced substantial growth, marked by a steady increase in publications and diverse thematic contributions since 2004. However, much of this expansion remains concentrated within Western institutions and journals, reflecting an uneven global landscape. Limited international collaboration and a dominance of single-country studies suggest barriers to broader knowledge exchange and inclusivity. Similarly, while the research output demonstrates strong academic grounding, the lack of interdisciplinary integration indicates that theoretical and methodological cohesion remains underdeveloped.

From a thematic standpoint, the keyword and clustering analyses indicate fragmentation within significant domains, including mental health, special education, and technological applications. Although the emergence of new areas, such as virtual reality, creativity, and systematic review approaches, signals methodological innovation, these remain loosely connected to core educational or therapeutic frameworks. The limited representation of media and technology in the literature further constrains understanding of how digital tools can enhance therapeutic engagement and educational accessibility. Strengthening links across these fragmented research clusters will be essential for building a coherent, evidence-based framework for music therapy in education.

Institutional and authorship analyses indicate that research productivity is heavily skewed toward a few universities and scholars, while most affiliations contribute only once. This suggests a significant degree of fragmentation and a low retention rate of active contributors in the domain. To address this problem, building capacity, creating mentorship networks, and making it easier for established and new researchers to work together are essential. The persistent shortage of qualified music therapists, coupled with funding and policy limitations, underscores the need for structural and institutional support to facilitate consistent implementation of music therapy programs in schools.

### 6.1 Key messages and practical implications

#### 6.1.1 For teachers

For Teachers: Findings highlight strong evidence linking music therapy to improved focus, emotional regulation, and inclusion, especially for learners with ADHD, autism, and anxiety. To improve engagement and classroom well-being, teachers should use structured musical interventions and work with certified therapists.

#### 6.1.2 For school administrators

The dominance of single-publication institutions indicates limited long-term engagement. Schools should establish sustained collaborations with universities and allocate dedicated spaces, time, and resources to embed music therapy consistently within curricula.

#### 6.1.3 For policymakers

Low international collaboration (6.18%) and Western research dominance suggest inequities in access and perspective. Policymakers should fund cross-cultural programs, enforce therapist certification standards, and include music therapy in national education and mental-health frameworks.

#### 6.1.4 For researchers

Subsequent research should amalgamate models such as CoMT, NMT, and Dalcroze, utilize longitudinal methodologies, and investigate virtual and data-driven therapeutic instruments to enhance theoretical and practical coherence.

## Data Availability

The original contributions presented in the study are included in the article/supplementary material, further inquiries can be directed to the corresponding author.
